# Sustainable innovative nanofibers containing Cobalt-MOF: a dual-action solution for microbial and chemical wastewater contamination

**DOI:** 10.3389/fchem.2025.1584064

**Published:** 2025-05-08

**Authors:** Safar M. Alqahtani, Dharmesh Sur, Ali Altharawi, R. Roopashree, Ahmed Hussein Zwamel, Taibah Aldakhil

**Affiliations:** ^1^ Department of Pharmaceutical Chemistry, College of Pharmacy, Prince Sattam Bin Abdulaziz University, Al-Kharj, Saudi Arabia; ^2^ Department of Chemical Engineering, Faculty of Engineering and Technology Marwadi University, Rajkot, Gujarat, India; ^3^ Department of Chemistry and Biochemistry, School of Sciences, JAIN (Deemed to be University), Bangalore, Karnataka, India; ^4^ Department of medical analysis, Medical laboratory technique college, the Islamic University, Najaf, Iraq; ^5^ Department of medical analysis, Medical laboratory technique college, the Islamic University of Al Diwaniyah, Al Diwaniyah, Iraq; ^6^ Department of medical analysis, Medical laboratory technique college, the Islamic University of Babylon, Babylon, Iraq

**Keywords:** cobalt-metal organic framework, poly vinyl alcohol, poly vinyl pyrrolidone, wastewater contaminants, Congo red, antimicrobial activity

## Abstract

Today, wastewater treatment is essential and inevitable due to the water crisis caused by climate change and population growth. Although numerous methods and synthetic compounds have been successful in practical and laboratory applications, developing novel multifunctional compounds remains of interest to scientists. For this purpose, a new nanofiber containing Cobalt-MOF (metal-organic framework), polyvinyl alcohol (PVA), and polyvinyl pyrrolidone (PVP), which are known environmentally friendly polymers, was synthesized. After characterization and structure determination, the nanofiber was investigated for its ability to adsorb Congo Red dye and inhibit known microbial species in wastewater. This study showed that 91% of the 400 mg/L Congo red solution was absorbed by 0.06 g/L of the synthesized Cobalt-MOF/PVA-PVP Nanofibers at 1 h. Additionally, seven well-known strains (including *Aeromonas hydrophila*, *Legionella pneumophila*, *Mycobacterium tuberculosis*, *Salmonella enterica*, *Pseudomonas aeruginosa*, *Escherichia coli*, and *Giardia lamblia*) in wastewater were inhibited (with MIC of 8 μg/mL to 64 μg/mL) to the extent that some antibiotics could not affect them. This performance of the newly synthesized nanofiber can be attributed to its physical, chemical, and structural characteristics, such as compounds with biological properties present in its structure, as well as its high specific surface area. Therefore, researching and synthesizing similar compounds using the method presented in this study can lead to their development and application in wastewater treatment processes.

## 1 Introduction

Pathogenic microbial agents such as *Aeromonas hydrophila*, *Legionella pneumophila*, *Mycobacterium tuberculosis*, *Salmonella enterica*, *Pseudomonas aeruginosa*, *Escherichia coli*, and *Giardia lamblia* can cause dangerous and sometimes fatal infections in humans. These microbia are predominantly found in sewage systems (LeChevallier et al., 2024). Due to population growth, climate change, and the scarcity of freshwater resources, there is an increasing need for adequate water purification methods for various applications, including industry, agriculture, and irrigation (Gude, 2017; Khondoker et al., 2023). Therefore, it is essential to remove pathogenic microbial and hazardous chemical pollutants from wastewater (Baig et al., 2021).

In addition to microbial pathogens, wastewater often contains chemical and industrial contaminants. One well-known hazardous chemical found in urban and industrial wastewater is dyes (Karri et al., 2021; Waheed et al., 2021). Congo Red, a highly water-soluble dye classified as a diazo dye, is considered a carcinogenic compound due to the diazo group in its structure (Siddiqui et al., 2023). Historically, Congo Red has been widely used in the textile industry and is currently employed in medical diagnostics, including amyloidosis (Samsami et al., 2020; Liang et al., 2022).

Although numerous synthetic compounds, such as nanoparticles, have been reported as antimicrobial agents or adsorbents for removing chemical pollutants, a compound that possesses both capabilities are particularly valuable (Rani et al., 2021; Ribeiro et al., 2022). Nanotechnology has emerged as a promising field for developing compounds with excellent antimicrobial properties (Hetta et al., 2023). Among these compounds are nanometal oxides, nanocomplexes, nanopolymers, metal-organic frameworks (MOFs), and nanofibers (Tang et al., 2021; Behyar et al., 2023).

Nanofibers are an important class of nanocomposites that not only have applications in pollutant absorption but also play significant roles in the medical industry as wound dressings and medical bandages (Homaeigohar and Boccaccini, 2020; El-Aswar et al., 2022; Parham et al., 2022). In synthesizing nanofibers, environmentally friendly polymers such as polyvinyl alcohol (PVA) and polyvinylpyrrolidone (PVP) are primarily used (Wu et al., 2022). Compounds with biological properties, such as plant extracts or metal-organic frameworks (MOFs), can be incorporated into nanofibers to enhance their activity (Kiadeh et al., 2021; Sharifi and Bahrami, 2024). This technique allows for the transfer of biological activity into a fibrous structure with a high specific surface area and high compressive and flexural strength (Zhou et al., 2020).

MOFs have attracted considerable attention from scientists due to their remarkable structures characterized by porosity and specific active surface areas (Zhang et al., 2022). Some reports indicate their antimicrobial and pollutant-absorbing activities (Adegoke et al., 2020; Pettinari et al., 2021). The choice of multidentate ligands and metals used in synthesis significantly influences their properties (Younis et al., 2021; Moharramnejad et al., 2023). Cobalt is one metal frequently reported in various MOFs due to its beneficial characteristics. Among the beneficial characteristics of cobalt MOF are its significant antibacterial, anticancer, and catalytic properties (Fan and Tahir, 2022; Li et al., 2022).

2,2′-Bipyridine-4,4′-dicarboxylic acid, which features two pyridine rings and two carboxylic acid groups, has been identified in several studies as an important ligand for producing MOFs (Li et al., 2023; Ren et al., 2024). Pyridine itself possesses numerous biological properties; thus, the presence of two pyridine units in 2,2′-bipyridine-4,4′-dicarboxylic acid enhances the bioactivity of compounds containing it (Allaka and Katari, 2023; Varshney and Mishra, 2023).

Given the importance of wastewater treatment processes for removing microbial pathogens and chemical pollutants, this study aims to synthesize a compound that combines these two functionalities. We utilized a mixture of polyvinyl alcohol (PVA) and polyvinylpyrrolidone (PVP) as environmentally friendly polymers, along with a metal-organic framework (MOF) containing 2,2′-bipyridine-4,4′-dicarboxylic acid, both of which are known for their antimicrobial and absorbent properties, to create a new type of nanofiber. We hypothesize that this newly synthesized fiber will exhibit a high capacity for absorbing Congo Red dye while effectively inhibiting and removing some of the most prevalent microbial agents found in wastewater, potentially rivaling some commercially available antibiotics.

## 2 Experimental

### 2.1 Materials and devices

Cobalt (II) nitrate hexahydrate (Merck) and 2,2′-bipyridine-4,4′-dicarboxylic acid (Sigma-Aldrich) were used in the synthesis of metal-organic frameworks (MOFs) via Ningbo Scientz Biotechnology Laboratory ultrasonic cleaner. Polyvinylpyrrolidone (130,000 g/mol, Sigma-Aldrich), polyvinyl alcohol (85,000 g/mol, Sigma-Aldrich), and acetic acid (Merck) were employed in the synthesis of nanofibers using an NE100 Single Nozzle electrospinning machine.

Congo Red (Merck) and a Labtronics L2T90 UV-Visible spectrophotometer were utilized in the absorption process.

Mueller-Hinton broth (Thermo Scientific), Mueller-Hinton Agar (Thermo Scientific), microbial strains obtained from the American Type Culture Collection (ATCC), and Labtronics L2T90 UV-Visible spectrophotometer (To prepare desired concentration of microbial suspension) was used in antimicrobial evaluations.

### 2.2 Synthesis method

#### 2.2.1 Synthesis of cobalt-MOF/PVA-PVP nanofibers

10 mmol Cobalt (II) nitrate hexahydrate and 10 mmol 2,2′-bipyridine-4,4′-dicarboxylic acid were added to 100 mL of double-distilled water and stirred until a homogeneous solution was obtained. The mixture was then subjected to ultrasonication at 300 W and 25°C. After synthesis, the metal-organic framework (MOF) compound was separated using nanofiltration and washed three times with ethanol, followed by three washes with double-distilled water. Finally, the compound was dried in an oven at 100°C for 1 hour under vacuum (Ahmad et al., 2022; Ramírez-Coronel et al., 2022).

A 20 mL solution of polyvinyl alcohol (PVA) and polyvinylpyrrolidone (PVP) was prepared in a 1:1 ratio using acetic acid, resulting in a concentration of 0.004%. Separately, a solution containing 0.01 mg of the metal-organic framework (MOF) was prepared by dispersing it in 25 mL of deionized water. The two solutions were then combined and stirred for 20 min at 80°C. Electrospinning were performed with a flow rate of 0.4 mL/h, a needle-to-collector distance of 22 cm, and an applied voltage of 28 kV. Finally, Cobalt-MOF/PVA-PVP Nanofibers was synthesized after the evaporation of the solvents (deionized water and acetic acid) at ambient temperature (Sargazi et al., 2019; Azizabadi et al., 2021; Moghaddam-manesh et al., 2022).

#### 2.2.2 Characterization

FT-IR spectra, SEM images, CHNO elemental analysis, XRD patterns, nitrogen adsorption/desorption isotherms, TGA curves, compressive strength, and flexural strength were used to identify and confirm the structure of the products. These analyses were obtained using equipment 1, 2, 3, 4, 5, 6, 7, and 8, respectively.

### 2.3 Congo red adsorption method

To determine the percentage of Congo red absorption, a specified quantity of V/BP-MOF was introduced into a Congo red solution in deionized water and mixed thoroughly at 200 rpm. The adsorption process was evaluated under various conditions, including adsorbent concentration, pH level, temperature, and duration of the adsorption. Subsequently, the absorbance was measured at 497 nm using a spectrophotometer (Moghaddam-Manesh et al., 2024).

### 2.4 Antimicrobial evaluation method

Clinical and Laboratory Standards Institute (CLSI) methods were used to investigate the antimicrobial activity against several microbial strains present in wastewater, such as *Aeromonas hydrophila*, *Legionella pneumophila*, *Mycobacterium tuberculosis*, *Salmonella enterica*, *Pseudomonas aeruginosa*, *Escherichia coli*, and *Giardia lamblia*. The strains were prepared at a concentration of 1 × 10^5^ CFU/mL in Mueller Hinton broth. This study investigated the Minimum Inhibitory Concentration (MIC) and Minimum Bactericidal Concentration (MBC) using microdilution and kill assays (Moghaddam-Manesh et al., 2020; Ramírez-Coronel et al., 2022).

## 3 Result and discussion

### 3.1 Synthesis and characterization of cobalt-MOF/PVA-PVP nanofibers

A new cobalt-MOF/PVA-PVP nanofibers was produced by electrospinning a mixture of PVP and PVA polymers.

**FIGURE 1:**
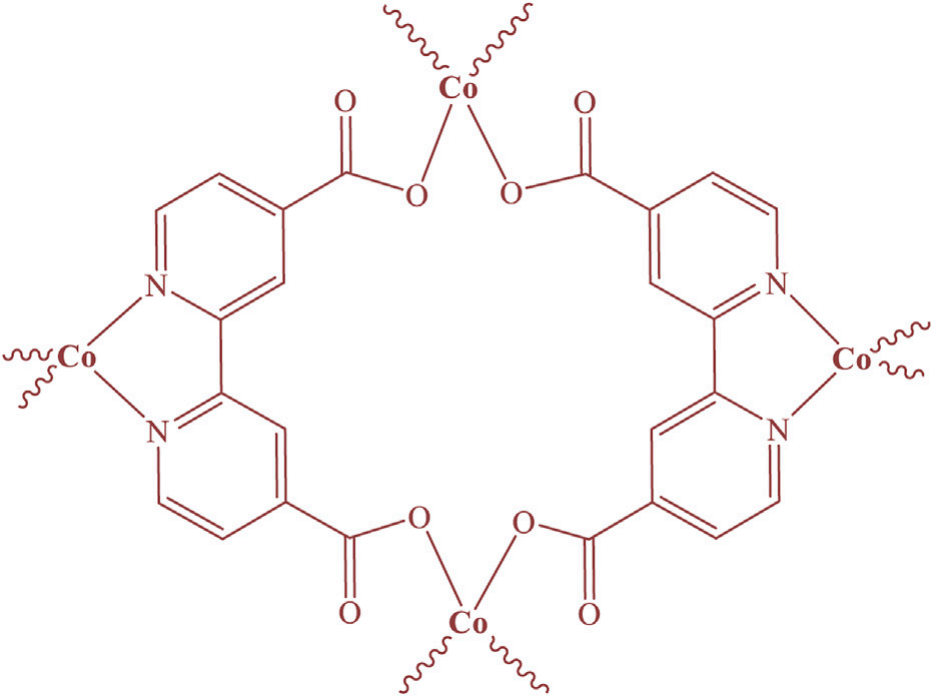
Structure of Cobalt-MOF.

Cobalt-MOF was synthesized from the reaction of Cobalt (II) nitrate hexahydrate and 2,2′-bipyridine-4,4′-dicarboxylic acid using ultrasonication. The structure of the synthesized MOF was proposed in Figure 1.

After confirming its structure, the electrospinning process was carried out, and the desired product (cobalt-MOF/PVA-PVP nanofibers) was synthesized and characterized by the necessary analyses.

The optimal conditions for nanofiber production, including the solvent ratio, the amount of metal-organic framework (MOF), temperature, flow rate, needle-to-collector distance, and applied voltage, were determined based on previous reports (Sargazi et al., 2019; Azizabadi et al., 2021).

For comparison, the analyses of cobalt-MOF and cobalt-MOF/PVA-PVP nanofibers are presented.

**FIGURE 2:**
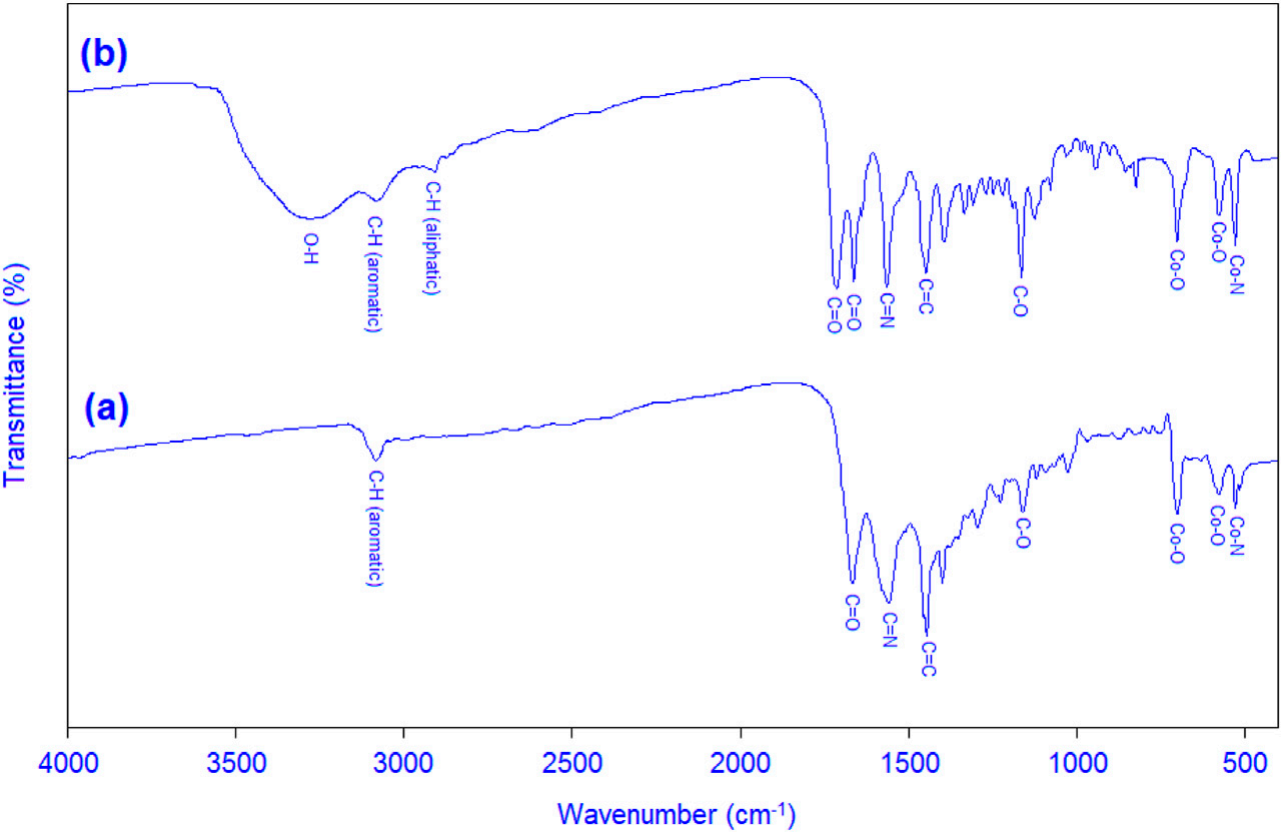
FT-IR spectra of cobalt-MOF **(a)**, cobalt-MOF/PVA-PVP nanofibers **(b)**.

FT-IR spectrum of cobalt-MOF (a) and cobalt-MOF/PVA-PVP (b) presented in Figure 2. In FT-IR spectrum of cobalt-MOF (a), cobalt-oxygen in the region of 560 cm^-1^, and 690 cm^-1^ (due to Co-ligand) (Ramírez-Coronel et al., 2022; Almeleebia et al., 2024), cobalt -nitrogen in the region of 530 cm^-1^ (due to Co-ligand) (Nayak et al., 2003), carbon-hydrogen aromatic in the region of 3,050 cm^-1^ (due to ligand), carbon = oxygen in the region of 1,650–1700 cm^-1^ (due to ligand), carbon = nitrogen in the region of 1,530 cm^-1^ (due to ligand), carbon = carbon aromatic in the region of 1,450 cm^-1^ (due to ligand), and carbon-oxygen in the region of 1,140 cm^-1^ (due to ligand) can be mentioned.

In FT-IR spectrum of cobalt-MOF/PVA-PVP (b), cobalt-oxygen in the region of 560 cm^-1^, and 690 cm^-1^ (due to Co-ligand) (Ramírez-Coronel et al., 2022; Almeleebia et al., 2024), cobalt -nitrogen in the region of 530 cm^-1^ (due to Co-ligand) (Nayak et al., 2003), oxygen-hydrogen in the region of 3,300 cm^-1^ (due to PVA), carbon-hydrogen aromatic in the region of 3,050 cm^-1^ (due to ligand), carbon-hydrogen aliphatic in the region of 2,900 cm^-1^ (due to PVA and PVP), two carbon = oxygen in the region of 1,650–1700 cm^-1^ (due to PVP and ligand), carbon = nitrogen in the region of 1,530 cm^-1^ (due to ligand), carbon = carbon aromatic in the region of 1,450 cm^-1^ (due to ligand), and carbon-oxygen in the region of 1,140 cm^-1^ (due to ligand) can be mentioned.

From the striking differences between FT-IR spectra of cobalt-MOF (a) and cobalt-MOF/PVA-PVP nanofibers (b) are the presence of several carbons = oxygen peaks, carbon-hydrogen aliphatic in the region of 2,900 cm^-1^, and oxygen-hydrogen in the region of 3,200 cm^-1^ in FT-IR spectrum of cobalt-MOF/PVA-PVP nanofibers.

Therefore, the binding and presence of cobalt-MOF in the final product can be confirmed.

**FIGURE 3:**
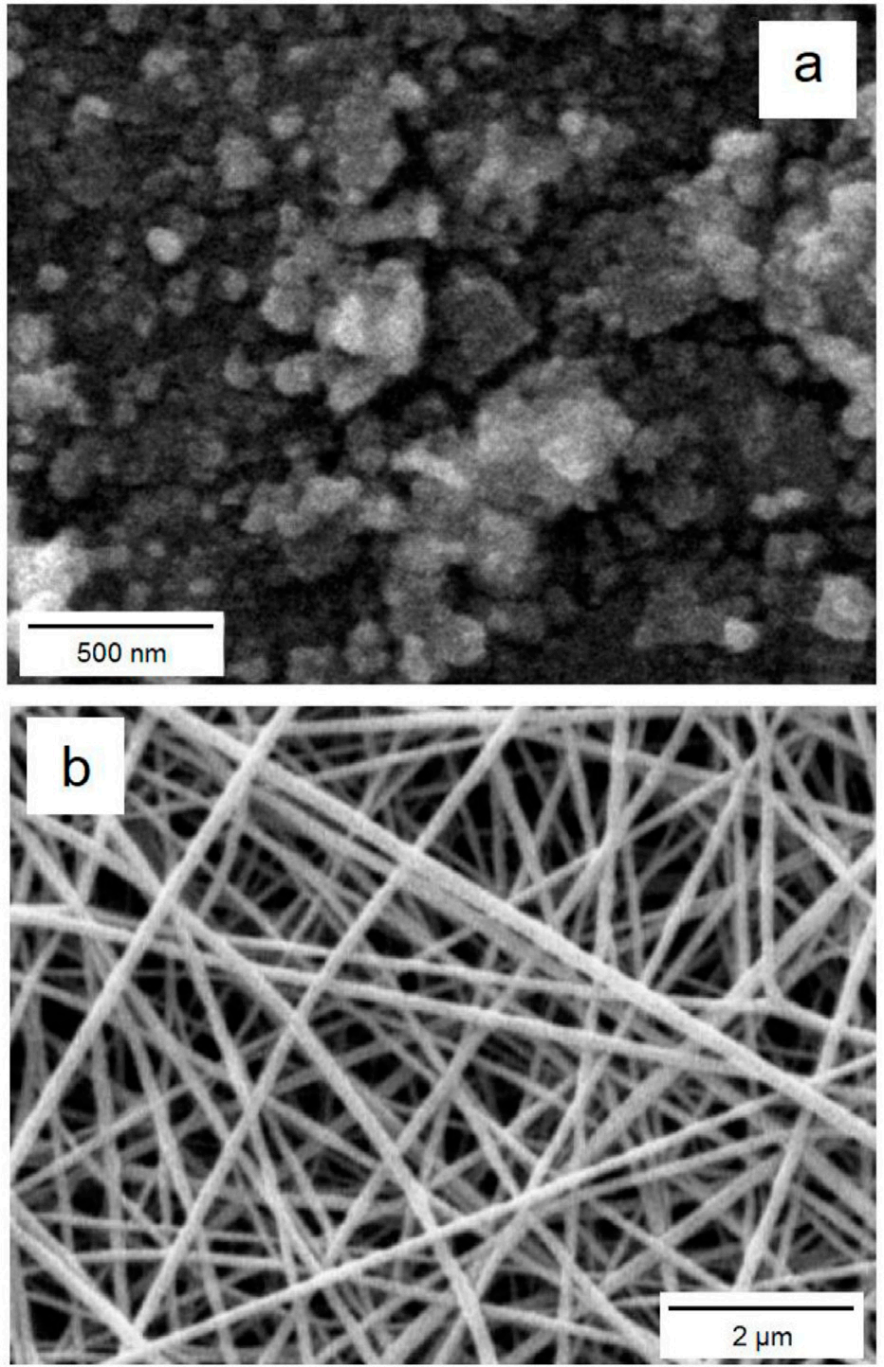
SEM images of cobalt-MOF **(a)**, cobalt-MOF/PVA-PVP nanofibers **(b)**.

The SEM images (Figure 3) of cobalt-MOF (a) and cobalt-MOF/PVA-PVP nanofibers (b) proved their nanosized as well as their uniform morphology.

Table 1 presents the results of CHNO elemental analysis for cobalt-MOF (a) and cobalt-MOF/PVA-PVP nanofibers (b). The percentages of elements, particularly hydrogen and carbon, in cobalt-MOF/PVA-PVP nanofibers have increased compared to those in cobalt-MOF. From the results, it can be concluded that other hydrocarbon groups are attached to cobalt-MOF, which can be used to prove the connection of PVA and PVP in the final product.

**TABLE 1:** CHNO elemental analysis of cobalt-MOF (a), cobalt-MOF/PVA-PVP nanofibers (b).

Element	Carbon (%)	Hydrogen (%)	Nitrogen (%)	Oxygen (%)
Compound				
A	45.72	4.65	6.53	15.18
B	58.29	8.91	7.42	16.76

**FIGURE 4:**
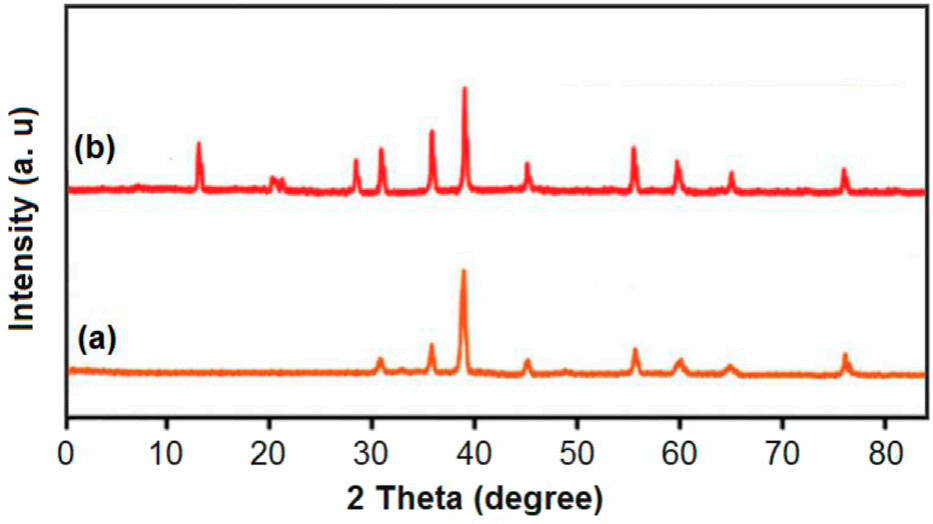
XRD pattern of cobalt-MOF **(a)** cobalt-MOF/PVA-PVP nanofibers **(b)**.

The peaks of the cobalt cubic structure corresponding to the planes 220, 311, 222, 400, 422, 511, 440, and 533 were observed at near 31°, 37°, 39°, 45°, 56°, 60°, 65°, and 77° (Lendzion-Bielun et al., 2013; Zawrah et al., 2016; Almeleebia et al., 2024) in the XRD (Figure 4) patterns of cobalt-MOF (a), and cobalt-MOF/PVA-PVP nanofibers (b). As can be seen in the XRD pattern of the final product, other peaks also appear, which are related to PVA (20°,29°) (Rajan et al., 2022) and PVP (12°, 21°) (Lv et al., 2019; Hu et al., 2021). Therefore, the presence of cobalt-MOF, PVA and PVP in the cobalt-MOF/PVA-PVP nanofibers can be proven.

**FIGURE 5:**
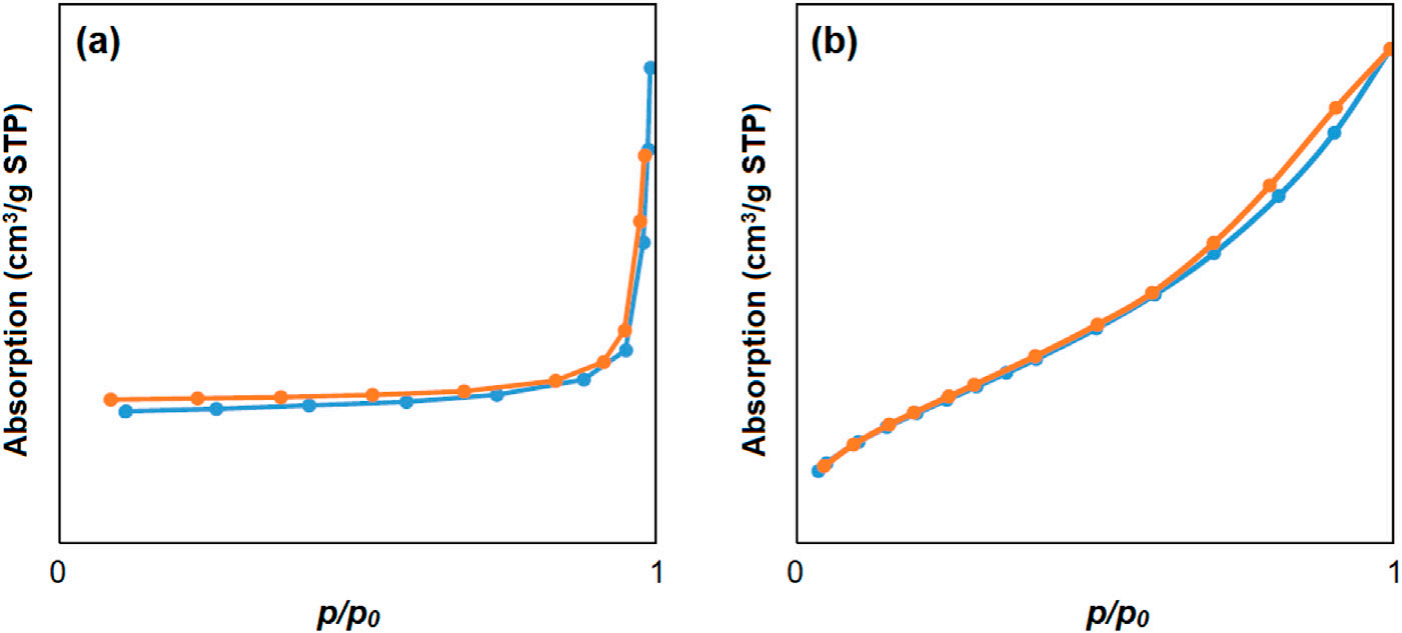
Nitrogen absorption/desorption of cobalt-MOF **(a)** cobalt-MOF/PVA-PVP nanofibers **(b)**.

The specific active surface area for cobalt-MOF (a), cobalt-MOF/PVA-PVP nanofibers (b) was calculated as 1742 m^2^/g and 2,415 m^2^/g, respectively, based on their nitrogen absorption/desorption behavior as shown in Figure 5. The nitrogen absorption/desorption of cobalt-MOFs is similar to type III and cobalt-MOF/PVA-PVP nanofibers is similar to type IV (Al-Ghouti and Da’ana, 2020). The pore volume of cobalt-MOFs and cobalt-MOF/PVA-PVP nanofibers were obtained as 0.004 cm^3^/g and obtained 0.007 cm^3^/g, respectively. Therefore, cobalt-MOF/PVA-PVP nanofibers exhibits the behavior of mesoporous materials and has higher porosity and specific surface area (Al-Ghouti and Da’ana, 2020). The placement of the cobalt-MOF in the nanofiber substrate through hydrogen bonding with PVA and PVP can be considered the reason for the high specific surface area of ​​the Cobalt-MOF/PVA-PVP nanofibers compared to Cobalt-MOF. According to previous studies, with increasing specific surface area, the contact of the compound with the factors or activity being studied increases. For example, with increasing specific surface area in the study of antimicrobial activity, the contact surface of the compound with microbial strains increases. Or in the study of dye adsorption activity, with increasing specific surface area, the contact of the compound with dyes increases. Therefore, higher properties of the compound are observed (Ghnim et al., 2025; Lyu et al., 2024; Mamman et al., 2024).

**FIGURE 6:**
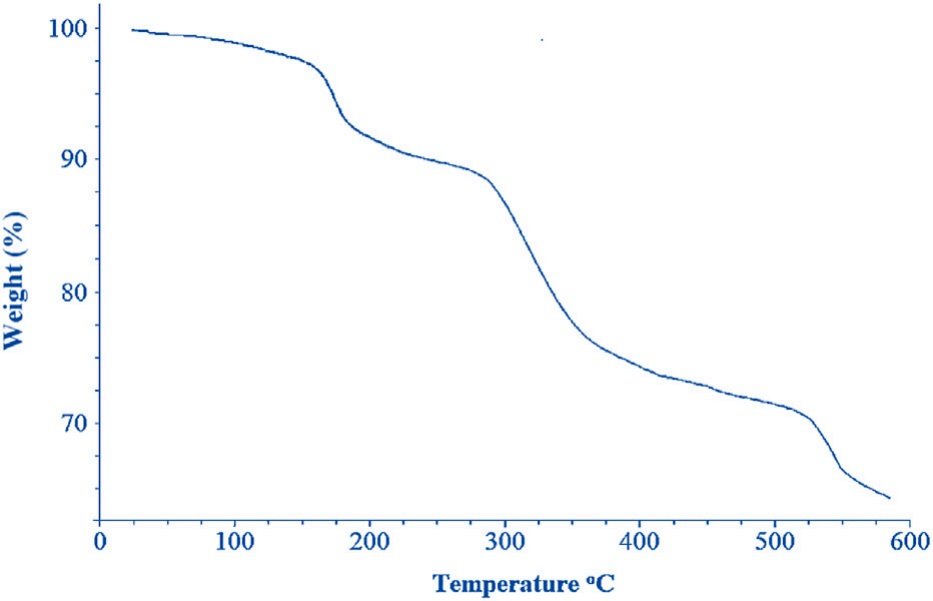
TGA curves of cobalt-MOF/PVA-PVP nanofibers.

The TGA curve (Figure 6) of cobalt-MOF/PVA-PVP nanofibers showed four weight loss at 147°C, 184°C, 320°C, and 519 ^°^C. The weight loss near 320°C and 519°C can be attributed to the decomposition of the 2,2′-bipyridine-4,4′-dicarboxylic acid and the destruction of the complex network. The other two significant weight losses in the final product at temperatures 147^°^C and 184°C, respectively, can be attributed to the decomposition of PVP and PVA.

**FIGURE 7:**
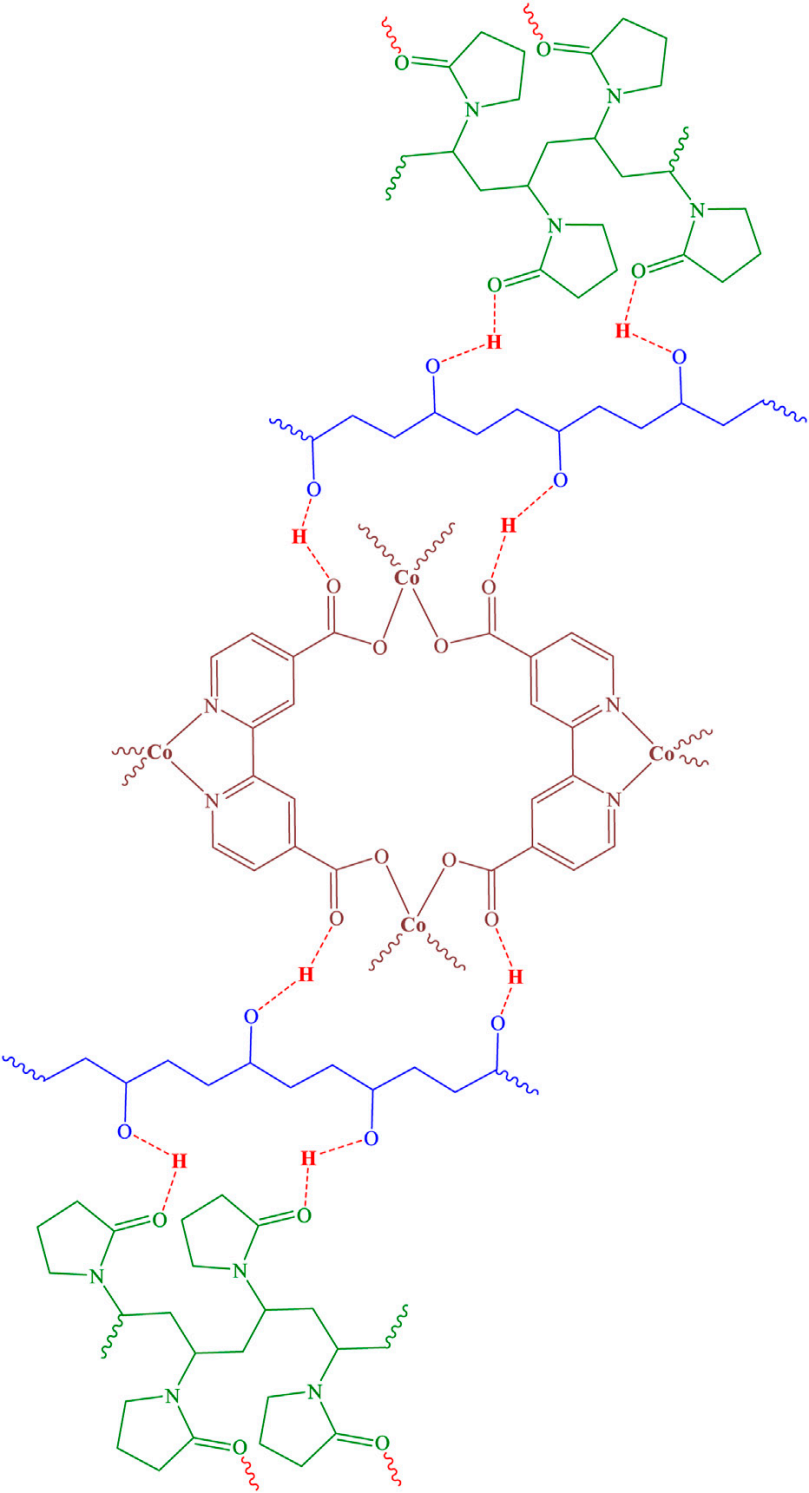
Structure of cobalt-MOF/PVA-PVP nanofibers.

Therefore, using the results obtained from the analyses, the structure of Figure 7 can be predicted for the cobalt-MOF/PVA-PVP nanofibers.

**FIGURE 8:**
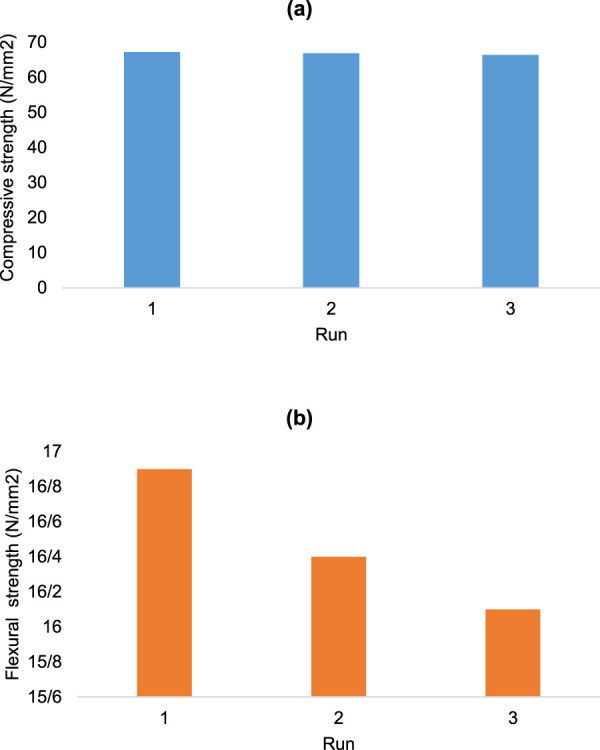
Compressive strength **(a)** and flexural strength **(b)** of cobalt-MOF/PVA-PVP nanofibers.

Compressive strength and flexural strength measurements (Figure 8) were the final analyses performed on the final product. The compressive strength (a) of the final product was 66.8 N/mm^2^ (n = 3) ± SD,) and the flexural strength (b) was 16.5 N/mm^2^ (n = 3) ± SD).

The compressive strength and flexural strength values of the final product were higher than those of some previously reported polymer blends that used PVA or PVP (Asiri et al., 2023; Khursheed et al., 2023). Therefore, it can be concluded that the mix of these two polymer blends resulted in increased compressive strength and flexural strength.

The results of the final product analysis proved that the nanofiber produced in this study is nano-sized, has a relatively high specific surface area and thermal stability, and has acceptable compressive strength and flexural strength. Therefore, the methods used in its production are acceptable.

The acceptable characteristics of the final product indicate its effectiveness in absorbing and inhibiting pathogenic strains. Therefore, the absorption properties of Congo red, one of the most important chemical agents in wastewater, as well as the ability to inhibit certain known pathogenic strains, were further tested on the final product.

### 3.2 Congo red adsorption of cobalt-MOF/PVA-PVP nanofibers

The high specific surface area, which increases the surface area of ​​the MOFs, can be introduced as one of the capabilities of these compounds in the adsorption process. In addition, the product structure and ability to create interactions are among the most important factors in the absorption process (Du et al., 2021; Huang et al., 2021). Based on previous studies, hydrogen bonding is an important factor in the adsorption process of Congo Red.

It was proven that the synthesized nanofiber in this study has a high specific surface area. Therefore, its performance in the Congo Red adsorption process was evaluated.

Several factors are effective in the adsorption process, the most important of which are the concentration of the adsorbed substance, the concentration of the adsorbent, the pH, the temperature, and the time of the adsorption process (Soliman and Moustafa, 2020). All of these factors were tested in the study of the adsorption of Congo Red by the cobalt-MOF/PVA-PVP nanofibers.

The percentage of Congo red adsorption was obtained in accordance with the equation described in Equation 1.

$$\text{Absorption }\left(\%\right)=\left[\left(I-R\right)\;/\;I\right]100$$ (1)

I: Initial concentration of Congo red (mg/L). R: Residual concentration of Congo red (mg/L).

Equation 1 Calculation of absorption (%).

For comparison, all tests were also performed on cobalt-MOF.

In the first step of investigating the adsorption of Congo Red by the final product, a fixed concentration of the final product was selected and tested on different concentrations of Congo Red. Factors such as pH, temperature, and time of the adsorption process were also kept constant.

For this purpose, 0.05 g/L of cobalt-MOF/PVA-PVP nanofibers were placed in solutions of 50, 100, 200, 400, 800, and 1,600 mg/L of Congo Red solution at pH seven and ambient temperature, and the adsorption process was studied for 1 h.

**FIGURE 9:**
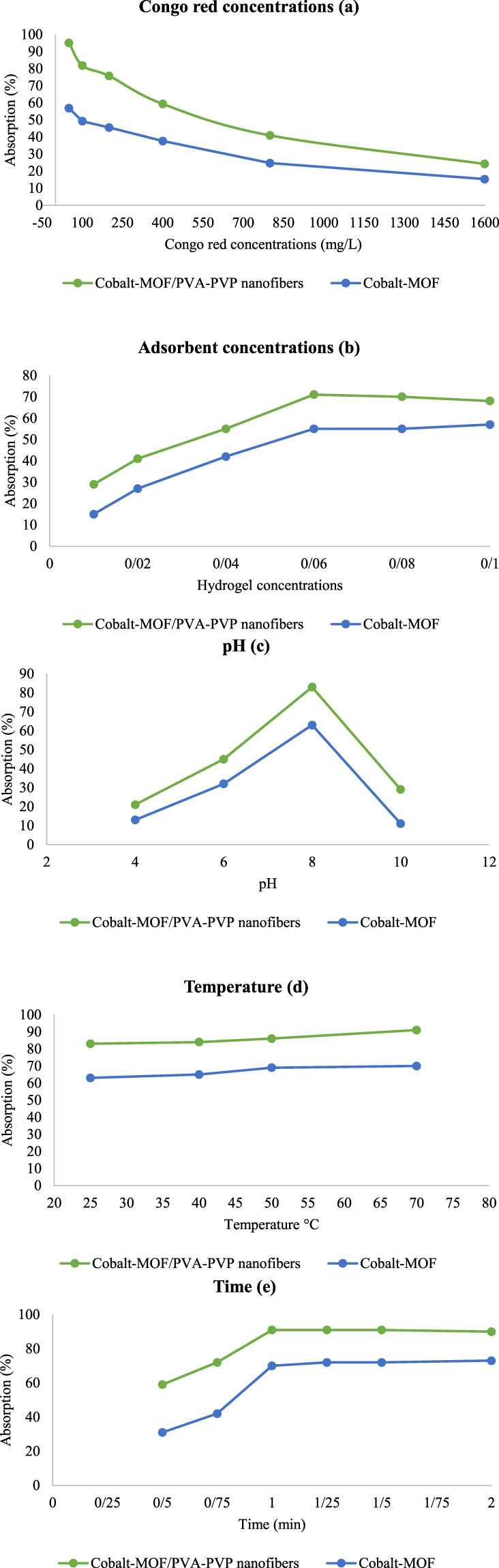
Congo Red absorption results in different conditions [Congo red concentrations **(a)** Adsorbent concentrations **(b)** pH **(c)** Temperature **(d)** Time **(e)**].

The results were completely selective, and it was determined that as the concentration of the Congo Red solution increased, the percentage of absorption decreased. So 95%, 82%, 76%, 59%, 41%, and 24% absorption was observed in Congo Red solution of 50, 100, 200, 400, 800, and 1,600 mg/L, respectively (Figures 9a).

The decrease in the adsorption percentage can be attributed to the saturation of the active adsorption sites of the adsorbent. One of these concentrations was designated as a fixed concentration, while other factors were tested and evaluated based on it. For this purpose, a concentration of 400 mg/L was chosen.

In evaluating the adsorbent concentration, concentrations of 0.01, 0.02, 0.04, 0.06, 0.08, and 0.1 g/L cobalt-MOF/PVA-PVP nanofibers were tested under the constant conditions of the previous step to absorb 400 mg/L of Congo Red solution.

The results obtained at concentrations of 0.01, 0.02, 0.04, 0.06, 0.08 and 0.1 g/L showed 29%, 41%, 55%, 71%, 70% and 68% absorption, respectively (Figures 9b).

The highest percentage of adsorption was observed at a concentration of 0.06 g/L and was therefore used as the optimal concentration in other experiments. The reason for the constant and reduced adsorption at concentrations higher than 0.06 g/L can be attributed to the aggregation of nanoparticles and, as a result, their accumulation and unavailability of active adsorption sites.

Strong and mildly acidic (4 and 6) and alkaline (8 and 10) pHs were another factor that was tested and evaluated in the process of absorbing a concentration of 400 g of Congo Red by 0.06 nanoparticles under constant temperature and time conditions according to the previous steps.

According to the results, the highest absorption occurred in mild alkaline conditions, with 83% observed at pH 8. At pHs 4, 6, and 10, the absorption was 21%, 45%, and 29%, respectively (Figures 9c).

At strongly acidic and alkaline pH levels, hydrolysis of the metal-ligand bond in the final product MOF leads to its degradation, resulting in reduced adsorption (Pramanik et al., 2023). At mildly acidic pH, the adsorption process is diminished due to the protonation of the nitrogen atoms in Congo Red and the oxygen atoms in the adsorbent, which reduces hydrogen bonding. In a mild alkaline environment, OH groups interact with the carbonyl groups of the adsorbent, increasing in the concentration of negative charge on the oxygen of the carbonyl group, ultimately leading to increased hydrogen bonding with Congo Red and increased adsorption (Guo and Bulin, 2021; Moghaddam-Manesh et al., 2024).

As the temperature increased from 25^°^C to 70°C, the absorption rate increased. At temperatures of 25, 40, 50, and 70°C, the absorption was 83%, 84%, 86%, and 91%, respectively (Figures 9d). This can be attributed to the increase in molecular movement (Wang et al., 2020).

In temperature tests, adsorption was also investigated at a concentration of 400 mg/L Congo Red solution by 0.06 g/L nanoparticles at pH eight for 1 h.

In the last tests, the adsorption process time was investigated under the above constant conditions. The adsorption process was tested at times of 0.5, 0.75, 1, 1.25, 1.5 and 2 h (Figures 9e). The highest adsorption occurred at 1 h, and its stability at 1, 1.25, 1.5, and 2 h can be attributed to the saturation of the active sites of the adsorbent.

**FIGURE 10:**
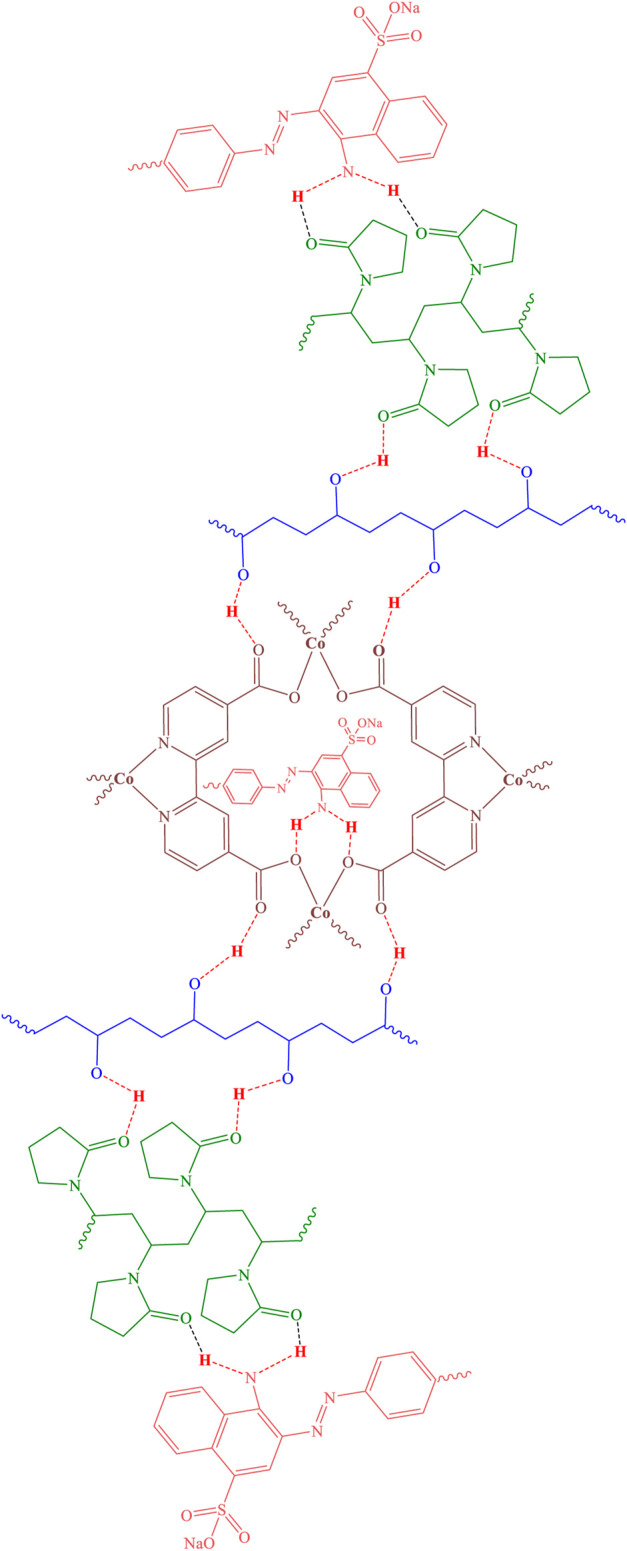
Congo Red absorption using cobalt-MOF/PVA-PVP nanofibers.

Figure 10 illustrates the process of Congo Red absorption by the final product.

As shown in the figure, the presence of multiple active sites in the final product, along with Congo Red, contributes to a high percentage of absorption.

As mentioned before, the high specific surface area of ​​the final product, along with its increased contact surface with Congo Red, is also very effective in this regard.

### 3.3 Antimicrobial activity of cobalt-MOF/PVA-PVP nanofibers

The antimicrobial activity of specific Cobalt-MOF/PVA-PVP Nanofibers was evaluated against various microbial strains present in wastewater, including strains *Aeromonas hydrophila (ATCC 7966)*, *Legionella pneumophila (ATCC 33152)*, *Mycobacterium tuberculosis (ATCC 27294)*, *Salmonella enterica (ATCC 35664)*, *Pseudomonas aeruginosa (ATCC 15442)*, *Escherichia coli (ATCC 25922)*, and *Giardia lamblia (ATCC 50803)*. The concentration of the nanofibers used in these studies ranged from 1 to 1,024 μg/mL, with a two-fold increase in concentration (e.g., 1, 2, 4, 8, 16, 32, up to 1,024). For comparison, biological evaluations were conducted on the synthesized Cobalt-MOF, PVA-PVP nanofibers without Cobalt-MOF, and amikacin.

The most significant result obtained from the study was the effectiveness of the nanofibers against all microbial strains tested. The Minimum Inhibitory Concentration (MIC) values for *Aeromonas hydrophila*, *Legionella pneumophila*, *Mycobacterium tuberculosis*, *Salmonella enterica*, *Pseudomonas aeruginosa*, *Escherichia coli*, and *Giardia lamblia* were determined to be 32 μg/mL, 32 μg/mL, 16 μg/mL, 8 μg/mL, 16 μg/mL, 32 μg/mL and 16 μg/mL, respectively.

The Minimum Bactericidal Concentration (MBC) values for *Aeromonas hydrophila*, *Legionella pneumophila*, *Mycobacterium tuberculosis*, *Salmonella enterica*, *Pseudomonas aeruginosa*, *Escherichia coli*, and *Giardia lamblia* were determined to be 32 μg/mL, 64 μg/mL, 32 μg/mL, 16 μg/mL, 16 μg/mL, 64 μg/mL and 32 μg/mL, respectively.

**FIGURE 11:**
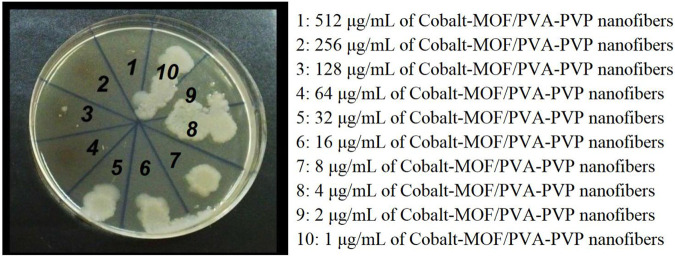
MBC of cobalt-MOF/PVA-PVP nanofibers (c) against *Escherichia coli* (ATCC 25922)

In Figure 11, as an example, the MBC image obtained from Cobalt-MOF/PVA-PVP Nanofibers (c) against *Escherichia coli* (ATCC 25922) is shown.

According to previous reports, cobalt and 2,2′-bipyridine-4,4′-dicarboxylic acid have strong antimicrobial properties. Combining them and producing Cobalt-MOF and Cobalt-MOF/PVA-PVP nanofibers containing them can lead to the production of compounds with unique antimicrobial properties (Mahadevi et al., 2022; Ameen and Majrashi, 2023). As the results proved, the effectiveness of Cobalt-MOF/PVA-PVP nanofibers was higher than that of Cobalt-MOF. In this regard, it can be pointed out that the specific surface area of ​​ Cobalt-MOF/PVA-PVP nanofibers is higher than that of Cobalt-MOF and as mentioned in Section 3.1. The increase in specific surface area leads to more contact of the studied microbial strains with the Cobalt-MOF/PVA-PVP nanofibers and consequently more contact of cobalt and 2,2′-bipyridine-4,4′-dicarboxylic acid (which have antimicrobial properties) (Ghnim et al., 2025; Lyu et al., 2024; Mamman et al., 2024).

The high specific surface area of the nanofibers is also crucial. This characteristic leads to increased contact with microbial strains and enhances the effectiveness of the Cobalt-MOF/PVA-PVP nanofibers compared to Cobalt-MOF.

Another notable finding was that the antimicrobial properties of the nanofibers were superior to those of amikacin (a well-known antibiotic). As shown in Table 2 of the results, amikacin had no effect on strains *Legionella pneumophila*, and *Giardia lamblia*, indicating microbial resistance. In contrast, the nanofibers demonstrated promising efficacy and adhesion.

**TABLE 2:** Antimicrobial activity results [Cobalt-MOF (a); PVA-PVP Nanofibers (b); Cobalt-MOF/PVA-PVP Nanofibers (c); Amikacin (d)].

Strains		Compounds			
		(a)	(b)	(c)	(d)
ATCC 7966	MIC	64	—	32	16
	MBC	128	—	32	32
ATCC 33152	MIC	64	—	32	—
	MBC	128	—	64	—
ATCC 27294	MIC	32	—	16	16
	MBC	32	—	32	32
ATCC 35664	MIC	64	256	8	4
	MBC	128	512	16	8
ATCC 15442	MIC	64	—	16	8
	MBC	256	—	16	16
ATCC 25922	MIC	64	128	32	4
	MBC	64	256	64	8
ATCC 50803	MIC	128	—	16	—
	MBC	128	—	32	—

MIC and MBC value: μg/mL.

In general, substances with antimicrobial activity and a high specific surface area in the structure of the Cobalt-MOF/PVA-PVP Nanofibers are important factors contributing to their high antimicrobial properties. The study underscores the potential of combining Cobalt-MOF with PVA-PVP to enhance antimicrobial effectiveness against resistant microbial strains.

## 4 Conclusion

Considering the importance of wastewater treatment and the elimination of pathogenic microbial agents and chemical contaminants, this study synthesized a new fiber containing Cobalt, 2,2′-bipyridine-4,4′-dicarboxylic, polyvinyl alcohol and, polyvinylpyrrolidone (Cobalt-MOF/PVA-PVP Nanofibers). The structure and characterization of the Cobalt-MOF/PVA-PVP Nanofibers were precisely confirmed using FT-IR spectra, SEM images, CHNO elemental analysis, XRD patterns, nitrogen adsorption/desorption isotherms, TGA curves, compressive strength, and flexural strength. In line with the research objectives, the effects of adsorption, particularly concerning Congo Red as a significant contaminant in wastewater, as well as the inhibition of microbial strains such as *Aeromonas hydrophila*, *Legionella pneumophila*, *Mycobacterium tuberculosis*, *Salmonella enterica*, *Pseudomonas aeruginosa*, *Escherichia coli*, and *Giardia lamblia*, were investigated using the Cobalt-MOF/PVA-PVP Nanofibers. The results demonstrated that the Cobalt-MOF/PVA-PVP nanofibers produced in this study possess biological properties and a high specific surface area of 2,415 m^2^/g, both of which are crucial for effective adsorption 91% of a 400 mg/L Congo Red solution was absorbed by 0.06 g/L of Cobalt-MOF/PVA-PVP nanofibers within 1 h and for the inhibition of microbial strains in wastewater, with a minimum inhibitory concentration (MIC) ranging from 8 μg/mL to 64 μg/mL. The main novelty of this study is to produce novel Cobalt-MOF/PVA-PVP Nanofibers with potential applications in removing chemical and microbial contaminants from wastewater. This indicates a strong capability for removing chemical and microbial contaminants from wastewater. Therefore, it is recommended that further studies focus on similar compounds.

## Data Availability

The original contributions presented in the study are included in the article/supplementary material, further inquiries can be directed to the corresponding author.
